# Successful pregnancy and delivery case in a peritoneal dialysis patient: A case report and review of literature

**DOI:** 10.1097/MD.0000000000047103

**Published:** 2026-01-09

**Authors:** Xueqing Hu, Hongqi Ren

**Affiliations:** a Department of Nephrology, Huaihai Hospital of Xuzhou Medical University, Xuzhou, China.

**Keywords:** delivery, peritoneal dialysis, pregnancy, uremia

## Abstract

**Rationale:**

Pregnancy in patients undergoing maintenance peritoneal dialysis (PD) is a rare and high-risk clinical scenario with limited documented successful outcomes, necessitating detailed reporting of management strategies.

**Patient concerns:**

A 26-year-old woman with end-stage renal disease on maintenance PD for over 5 years presented with an incidentally discovered viable singleton pregnancy at 26⁺ weeks of gestation.

**Diagnoses:**

Chronic kidney disease (CKD), Stage 5D (on PD). Pregnancy, singleton, at 26⁺ weeks of gestation. Severe anemia, associated with CKD. Secondary hyperparathyroidism, severe. Hypertension, in the context of CKD.

**Interventions:**

An individualized PD regimen (reduced the amount of dialysis per session, increased exchange frequency) was implemented. Comprehensive management included correction of anemia (transfusions, erythropoiesis-stimulating agents, iron supplements), antihypertensive therapy, and control of hyperparathyroidism. Close multidisciplinary surveillance was maintained. Emergency cesarean section was performed at 30⁺ weeks due to fetal distress, preceded by a single hemodialysis session.

**Outcomes:**

A live infant (1100 g) was delivered, requiring neonatal intensive care but demonstrating favorable long-term growth and neurodevelopment. The maternal postoperative course was complicated by an intra-abdominal infection associated with the PD catheter, which resolved following its removal.

**Lessons:**

Successful pregnancy in PD patients requires meticulous dialysis prescription tailoring, aggressive management of comorbidities, and sustained multidisciplinary collaboration. Postpartum PD catheter removal should be considered to mitigate infectious risk. Favorable maternal and neonatal outcomes are achievable with dedicated, individualized care.

## 1. Introduction

Recently, chronic kidney disease (CKD) is increasing in incidence in China.^[1]^ CKD in women of reproductive age poses significant challenges to achieving successful pregnancy. Among dialysis-dependent patients, peritoneal dialysis (PD) has historically been considered a relative contraindication for pregnancy due to physiological constraints and high fetal-maternal risks.^[2]^ This case report aims to detail the clinical journey and treatment considerations in managing a patient with this rare pregnancy and delivery case in a PD patient. This may provide some reference for the pregnancy management of future PD patients.

## 2. Case description

### 2.1. History of present illness

A 26-year-old woman was admitted on December 18, 2023, for adjustment of her dialysis regimen after a routine follow-up examination revealed a single live fetus at 26⁺ weeks of gestation.

The patient developed systemic edema with wheezing, vomiting and poor appetite in May 2018 without obvious cause. The patient was initially diagnosed with uremia at an outside hospital, and PD was initiated after PD catheter insertion surgery (specific details unknown). In August 2018, she was transferred to our hospital for regular PD treatment, and the peritoneal equilibration test showed lower than average solute transport, then she was commenced on continuous ambulatory PD, and the dialysis prescription was 1.5% PD solution with 2 exchanges daily of 2000 mL, 2.5% PD solution with 1 exchange daily of 2000 mL, with dwell time for 4 hours each time; The ultrafiltration volume was around 600 to 1200 mL/d, and the urine volume was around 800 to 1400 mL/d. At the same time, erythropoietin (EPO) was used to treat anemia, and nifedipine tablets were used to control blood pressure. In September 2019, the patient’s clinical parameters were reassessed. Her ultrafiltration volume had decreased to approximately 400 to 600 mL/day, while her urine volume remained around 800 to 1300 mL/day. Relevant laboratory values were as follows: hemoglobin (Hb) 109 g/L, blood urea nitrogen (BUN) 10.50 mmol/L, serum creatinine (SCr) 476.3 μmol/L, calcium (Ca²⁺) 2.16 mmol/L, phosphorus (P³⁻) 0.95 mmol/L, and parathyroid hormone (PTH) 358.9 pg/mL. In response, the dialysis prescription was switched to 2 exchanges daily of 2000 mL 1.5% PD solution and 2 exchanges daily of 2000 mL 2.5% PD solution. This adjustment resulted in improved ultrafiltration, with a daily volume ranging between 700 and 1100 mL, and the dialysis adequacy index (Kt/V) was maintained at 1.5 to 1.7. On April 24, 2021, the Hb levels were rechecked at 100 g/L, and EPO was switched to Roxadustat capsules to improve anemia. On August 24, 2021, the PTH level was elevated to 1877.8 pg/mL, she was given cinacalcet combined with paricalcitol to treat secondary hyperparathyroidism. Recheck PTH decreased to 946 pg/mL. In early December 2023, it was discovered that her weight had increased, and the abdomen was bulging compared to before. So she came to our outpatient department for a follow-up examination, and the color Doppler ultrasound examination showed a single live fetus in the uterus, considering the possibility of combining pregnancy, the patient was admitted to the hospital on December 18, 2023, to adjust the dialysis prescription and observe the condition of the fetus. During the course of the disease, the patient’s mental state, diet, and sleep are good. The urine volume was about 800 to 1000 mL, and the stool was normal.

### 2.2. Medical history

She had a history of hypertension for 5 years, with a maximum of 180/120 mm Hg. Nifedipine sustained-release tablets and labetalol hydrochloride tablets were given to control blood pressure, which is currently controlled at around 140/110 mm Hg.

### 2.3. Marriage and childbearing history

She got married at the age of 19, and had 1 miscarriage in 2016; Irregular menstrual cycle, menarche at the age of 14, menstruation lasting 4 to 5 days with an interval of 15 to 60 days, last menstrual period unknown, no dysmenorrhea, normal menstrual flow, and no blood clots.

Personal history and family history are not special.

### 2.4. Physical examination

On admission, the patient was alert and in a fair general condition.

Vital signs: Temperature 36.5°C, heart rate 82 beats/min, respiratory rate 20 breaths/min, blood pressure 154/86 mm Hg.

General appearance: Ill-looking, with pale conjunctiva (anemic appearance).

Cardiovascular: Regular heart rhythm, no audible murmurs, gallops, or rubs.

Pulmonary: Clear breath sounds bilaterally, with no wheezes or crackles.

Abdomen: Distended, consistent with advanced pregnancy and the presence of dialysate. The PD catheter was in situ and well-secured. No tenderness or guarding was noted. Fetal heart rate was approximately 150 beats/min (auscultated).

Extremities: No peripheral edema was observed.

### 2.5. Laboratory examination

Blood routine: White blood cell (WBC) 11.36 × 10^9^/L, neutrophil percentage 74.4%, Hb 59g/L, Platelets 246 × 10^9^/L, high-sensitivity C-reactive protein (CRP) 3.32 mg/L.

Blood biochemistry:Bicarbonate (HCO_3_^−^) 20.50 mmol/L, BUN 20.90 mmol/L, SCr 562.65 μmol/L, K^+^ 3.50 mmol/L, Ca^2+^ 2.31 mmol/L, P^3−^ 1.70 mmol/L, PTH 3135.24 pg/mL.

**Cardiac biomarkers:** Troponin I < 0.01 ng/mL, N-terminal pro brain natriuretic peptide 8183.38 pg/mL.

**Peritoneal dialysis fluid routine test:** transparent, protein (−), red blood cell microscopy: 20/μL, WBC microscopy: 10/μL. Biochemistry: total protein 0.471 mg/mL, glucose 37.9 mmol/L, chloride 99 mmol/L.

### 2.6. Supplementary examination

Gynecological ultrasound: A single viable fetus is present in the uterine cavity. The fetal size is approximately at 26^+^ weeks of gestation. The amniotic fluid is excessive.

Cardiac ultrasound: Left ventricular ejection fraction: 65%, aortic valve regurgitation (mild), mitral valve regurgitation, tricuspid valve regurgitation (mild), normal left ventricular systolic function.

Diagnoses on Admission: CKD, stage 5D (on PD). Pregnancy, singleton, at 26⁺ weeks of gestation. Severe anemia, associated with CKD. Secondary hyperparathyroidism, severe. Hypertension, in the context of CKD.

Treatment process and follow-up: After admission, based on the patient’s medical history and ultrasound examination, it was found that at 26^+^ weeks of pregnancy, the abdominal circumference increased and the intra-abdominal volume decreased. The dialysis prescription was adjusted to reduce the amount of dialysis per session and increase the number of dialysis sessions, that was, 1500 mL of 1.5% PD solution per session, twice a day; 2.5% PD solution 1500 mL/time, 3 times a day. At the same time, she was treated with transfusions of red blood cell suspension, EPO, iron supplements and folic acid to correct anemia, improving calcium and phosphorus metabolism with palicalcitriol, and controlling blood pressure with nifedipine sustained-release tablets combined with labetalol hydrochloride tablets. The patient’s urine output increased and remained at around 1200 to 1600 mL/d, with an ultrafiltration volume of around 400 to 600 mL/d. The BUN was rechecked at 17.95 mmol/L, SCr 588.30 μmol/L. After the condition stabilized, she was discharged on December 29, 2023. On January 4, 2024, the patient’s reexamination at Xuzhou Maternal and Child Health Hospital showed that the fetal heart rate was good, but the ultrasound showed that the ratio of systolic to diastolic pressure in the fetal umbilical artery was high (5.27).

On January 14, 2024, she was readmitted to our department due to high blood pressure and chest tightness. As the patient’s abdominal circumference continued to increase, the dialysis regimen was adjusted to 1.5% peritoneum dialysis fluid 1000 mL/time, twice/day, 2.5% PD fluid 1000 mL/time, 3 times/day, ultrafiltration volume was about 1000 mL/day, but urine volume was reduced to 200 to 500 mL/d. Her Hb was found to be 79 g/L, BUN 25.80 mmol/L, SCr 65.85 μmol/L, Ca^2+^ 2.45 mmol/L, P^3-^ 2.30 mmol/L, and PTH 2932.22 pg/mL. Continued symptomatic treatment included blood pressure control, correction of anemia, and improvement of hyperparathyroid (Fig. S1, Supplemental Digital Content, https://links.lww.com/MD/R113). The Patient regularly monitor fetal heart rate and fetal development in obstetrics outpatient department.

On January 16, 2024, the patient complained of reduced fetal movement, and emergency examination showed that the fetal movement was significantly reduced compared with before, and fetal heart monitoring indicated a nonreactive type, with a heart rate of about 150 times/min, suggesting intrauterine hypoxia a risk of intrauterine death at any time. After multidisciplinary consultation, it was decided to perform a lower segment cesarean section. Before the operation, a temporary internal jugular venouseter was placed for hemodialysis (HD) once, and low molecular weight heparin sodium 1500 IU was used for anticoagulation. She was administered general anesthesia, and the surgical process proceeded smoothly, the newborn Apgar score: 5 points in 1 minute, 9 points in 5 minutes, weight 1100 grams. The PD catheter was retained and an abdominal drainage tube was placed during the operation at the patient’s request. After the operation, the patient was transferred to the intensive care unit (ICU) for continuous renal replacement therapy, infection prevention, and tocin control of uterine bleeding, and other supportive measures. The patient’s vaginal lochia and abdominal drainage were both reduced.

On January 18, the patient was transferred from ICU to our department and continued to receive HD 3 times a week, 4% Trisodium citrate was used for anticoagulation. On January 20, the patient’s body temperature fluctuated between 36.5℃ and 37.5℃, WBC 26.79 × 10^9^/L, CRP 161.04 mg/L, the amount of abdominal drainage fluid has increased compared to before, with a maximum drainage of 700 mL/24h of hemorrhagic fluid. Biapenam was added for anti infection, and we removed the abdominal drainage tube on January 23, the abdominal cavity was then flushed with 2000 mL of 1.5% PD solution through a PD catheter (once a day). On January 25, the patient’s highest body temperature reached 39.4℃, and the peritoneal drainage fluid was turbid, the routine examination of the dialysate showed: red blood cells 33,000/μL, WBCs 14,896/μL. Multiple blood cultures and dialysate cultures were negative, abdominal ultrasound showed local abdominal wall muscle layer edema in the left middle and lower abdomen. Anti infection and nutrition improvement treatments were continued, and the body temperature fluctuated at 37.5 ℃ to 38 ℃. The PD catheter was removed on February 7, and a culture of the catheter tip was performed, which was negative. The patient’s body temperature gradually returned to normal, and the blood WBC decreased to 10.24 × 10^9^/L, CRP 49.92 mg, and the patient was discharged on February 15, 2024. The patient regularly underwent HD treatment after discharge. At present, both the patient and the newborn are recovering well.

### 2.7. Newborn outcome and follow-up

The newborn, delivered prematurely at 30^+^ weeks with a birth weight of 1100 g, was admitted to the neonatal ICU. The infant’s hospital course included management for respiratory distress syndrome, jaundice, and establishment of enteral nutrition. After a prolonged neonatal ICU stay, the baby was discharged in stable condition. At a corrected gestational age of 18 months, the child weighed 10 kg and demonstrated age-appropriate neurodevelopmental progress, indicating favorable long-term growth and development following a very preterm birth.

## 3. Discussion

Pregnancy in patients with end-stage renal disease on dialysis is an uncommon and high-risk clinical scenario. Here, we conducted a review of relevant literature (as shown in Table S1, Supplemental Digital Content, https://links.lww.com/MD/R113). For example, the first documented case of pregnancy in a PD patient was reported by Cattran and Benzie in 1983, although the pregnancy ended in fetal loss at 30 weeks of gestation.^[3]^ A recent French retrospective study (2006–2020) further highlighted the rarity of pregnancy in PD, with only 2.9% of 348 pregnancies in women on maintenance dialysis occurring in PD patients.^[4]^ In China, reports of successful pregnancy and delivery in PD patients are exceedingly scarce.

Theoretically, PD offers several potential advantages over HD in pregnancy, including greater hemodynamic stability, continuous solute and fluid removal, avoidance of systemic anticoagulation, and better preservation of residual renal function.^[3,5,6]^ However, as gestation advances, the expanding uterus reduces intra-abdominal volume and effective peritoneal surface area, often necessitating adjustments in dialysis prescription. Common strategies include reducing dwell volumes and increasing exchange frequency to maintain adequacy while minimizing abdominal discomfort and compromising ultrafiltration.^[2]^ Our patient’s regimen was similarly modified multiple times during pregnancy to accommodate these physiological changes.

Pregnant women on dialysis face significantly elevated risks of maternal and fetal complications, including preeclampsia, anemia, polyhydramnios, preterm birth, intrauterine growth restriction (IUGR), and small-for-gestational-age infants.^[3,5,7,8]^ Preterm delivery and low birth weight are particularly common. A meta-regression analysis by Piccoli et al reported a higher incidence of small-for-gestational-age infants in PD compared to HD (67% vs 31%), with a median gestational age of 34 weeks and mean birth weight of 1780 g in PD pregnancies.^[9]^ In the present case, emergency cesarean section was performed at 30^+^ weeks due to fetal distress and intrauterine hypoxia, resulting in a birth weight of 1100 g, consistent with these earlier observations. Elevated maternal BUN is thought to contribute to polyhydramnios and preterm labor through fetal osmotic diuresis.^[2,10]^ Moreover, reduced dialysate efficacy in late pregnancy due to mechanical constraints may further exacerbate uremia, increasing the risk of adverse outcomes.

PD-specific complications during pregnancy may include peritonitis, hemoperitoneum, catheter malfunction, and abdominal discomfort.^[2,6,11–14]^ There is no consensus on the optimal PD prescription during pregnancy. Some experts recommend using low-glucose concentration solutions (1.5% or 2.5%), increasing exchange frequency, and reducing dwell volumes to 500 to 1000 mL per exchange to minimize intra-abdominal pressure.^[15]^ Hou et al suggested a daily dialysate volume of 1.5 to 3 L administered over 4 or more exchanges, with dwell times of 4 to 8 hours.^[2]^ Adequacy targets are also debated, while a BUN level < 17.85 mmol/L (<50 mg/dL) is often proposed as a goal,^[16]^ its reliability is limited by variable urea generation from both mother and fetus. Kt/V has been proposed as an alternative metric, with a target of 2.2 to 2.4 suggested in some studies,^[2]^ though evidence remains limited. In our patient, despite aggressive prescription adjustments, toxin levels rose in the third trimester, prompting consideration of transition to HD. However, due to acute fetal distress, only a single HD session was performed prior to emergent delivery.

The management of pregnancy in dialysis patients requires early and continuous multidisciplinary collaboration involving nephrologists, obstetricians, neonatologists, and dietitians.^[4,17–19]^ Shared decision-making is critical, particularly regarding pregnancy continuation, given the high rates of elective termination-up to 29.9% in 1 study.^[4]^ Therefore, multidisciplinary collaboration, including experienced and professional multidisciplinary diagnosis and treatment of nephrologists, obstetricians, nutritionists, pediatricians, is particularly important during pregnancy in PD patients.^[6,20–23]^ Ethical considerations, including patient autonomy, fetal anomaly screening, and long-term maternal health, must be carefully weighed.^[24,25]^ In this case, the patient opted to continue the pregnancy despite medical advice to the contrary, underscoring the importance of respecting patient values while providing comprehensive support.^[26]^

Postpartum management of the PD catheter also warrants attention. In this case, the catheter was retained at the patient’s request but was associated with postoperative intra-abdominal infection that resolved only after catheter removal. This suggests that early catheter removal after delivery may be prudent to avoid infectious complications.

Notably, the patient’s urine volume increased during mid-pregnancy but declined later, possibly reflecting initial increases in renal perfusion followed by compressive effects of the gravid uterus. This dynamic highlights the importance of monitoring residual renal function throughout gestation.

A limitation in this case was the unavailability of metagenomic next-generation sequencing (mNGS) for diagnosing abdominal infection. mNGS offers broad pathogen detection, high sensitivity, and rapid turnaround, making it particularly useful in culture-negative cases or patients with prior antibiotic exposure.^[27,28]^ Its application could have guided targeted antimicrobial therapy earlier in the clinical course.

## 4. Conclusion

This case of PD pregnancy patient has many problems and risks during pregnancy, including the adjustment of dialysis scheme in the late stage of pregnancy, treatment of anemia and hypertension, the management of abdominal infection after delivery, and the rescue of premature infants, etc. We achieved better maternal and infant outcomes through individualized adjustment of dialysis and treatment plans, and strengthened collaboration among multidisciplinary teams. Therefore, the diagnosis and treatment process of this case provides some experience and lessons for the management of pregnancy in PD patients.

## Acknowledgments

The authors thank the patient for granting permission to publish this manuscript. This work was supported by the Medical Science and Technology Innovation Project of Xuzhou Municipal Health Commission (XWKYHT20230075).

## Author contributions

**Data curation:** Xueqing Hu.

**Funding acquisition:** Xueqing Hu.

**Resources:** Hongqi Ren.

**Writing – original draft:** Xueqing Hu.

**Writing – review & editing:** Hongqi Ren.

## Supplementary Material


