# Supplementary material for: Successful pregnancy and delivery case in a peritoneal dialysis patient: A case report and review of literature
Source: Medicine (Baltimore). 2026 Jan 9;105(2):e47103. doi: 10.1097/MD.0000000000047103 (PMC12794962; doi:10.1097/MD.0000000000047103)
Supplement: Supplementary file 1 [file medi-105-e47103-s001.pdf]

Supporting Information(SI)

Fig legend:

Figure S1 Changes of Hb and Hct before and after pregnancy

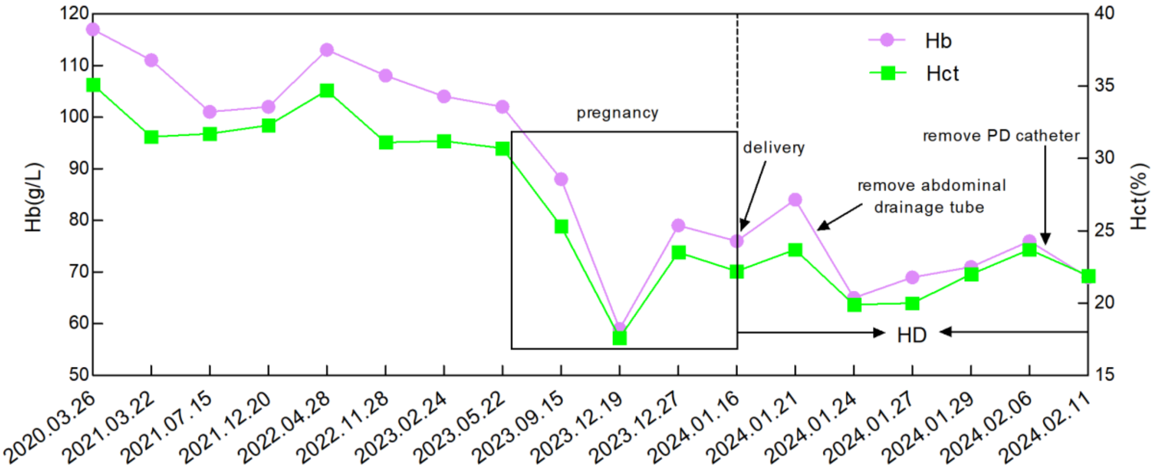

Figure S1 Changes of Hb and Hct before and after pregnancy

Data are presented for a single patient from the pre-pregnancy period through the third trimester. Data points represent mean values from each trimester. Hb=hemoglobin, Hct=Hematocrit.

Supporting Information(SI)

Table legend:

Table S1 Summary of Published Cases of Successful Pregnancy in Peritoneal Dialysis Patients

Study / Case Report	Country	PD Regimen Adjustments	Key Management Strategies
Cattran & Benzie (1983) [a]	Canada	Not specified	First reported case; highlighted high risks
Smith et al. (2005) [b]	USA	dwell volume, increased frequency	Multidisciplinary care, nutritional support
Piccoli et al. (2016) [c]	Multi-country	Varied regimens	Meta-analysis highlighting PD-specific outcomes
Lim et al. (2017) [d]	Malaysia	Low-volume exchanges, increased frequency	Close fetal monitoring, hypertension management
Veríssimo et al. (2022) [e]	Portugal	Reduced fill volume, frequent exchanges	Individualized dialysis, anemia correction
Current Case (2024)	China	Progressive volume reduction, increased frequency	Dynamic PD adjustments, multidisciplinary care

Summary of selected published cases of successful pregnancy in patients on peritoneal dialysis (PD). PD = peritoneal dialysis .

References

[a] Cattran DC, Benzie RJ. Pregnancy in a continuous ambulatory peritoneal dialysis patient. *Perit Dial Bull.* 1983;3:13-4.

[b] Smith WT, Darbari S, Kwan M, et al. Pregnancy in peritoneal dialysis: a case report and review of adequacy and outcomes. *Int Urol Nephrol.* 2005;37:145-51.

[c] Piccoli GB, Minelli F, Versino E, et al. Pregnancy in dialysis patients in the new millennium:a systematic review and meta-regression analysis correlating dialysis schedules and pregnancy outcomes. *Nephrol Dial Transplant.* 2016;31:1915-34.
[d] Lim TSC, Shanmuganathan M, Wong I, et al. Successful multigravid pregnancy in a 42-year-old patient on continuous ambulatory peritoneal dialysis and a review of the literature. *BMC Nephrol.* 2017;18:1-5.
[e] Veríssimo R, Nogueira E, Bernardo J, et al. Pregnancy in a woman undergoing peritoneal dialysis: Management and dialysis options. *Clin Nephrol Case Stud.* 2022;10:32.
